# Phylogeography on the rocks: The contribution of current and historical factors in shaping the genetic structure of *Chthamalus montagui* (Crustacea, Cirripedia)

**DOI:** 10.1371/journal.pone.0178287

**Published:** 2017-06-08

**Authors:** Federica G. Pannacciulli, Ferruccio Maltagliati, Christian de Guttry, Yair Achituv

**Affiliations:** 1 ENEA—Marine Environment Research Centre, La Spezia, Italy; 2 Department of Biology, University of Pisa, Pisa, Italy; 3 The Mina and Everard Goodman Faculty of Life Sciences, Bar Ilan University, Ramat-Gan, Israel; Universita degli Studi della Tuscia, ITALY

## Abstract

The model marine broadcast-spawner barnacle *Chthamalus montagui* was investigated to understand its genetic structure and quantify levels of population divergence, and to make inference on historical demography in terms of time of divergence and changes in population size. We collected specimens from rocky shores of the north-east Atlantic Ocean (4 locations), Mediterranean Sea (8) and Black Sea (1). The 312 sequences 537 bp) of the mitochondrial cytochrome *c* oxidase I allowed to detect 130 haplotypes. High within-location genetic variability was recorded, with haplotype diversity ranging between *h* = 0.750 and 0.967. Parameters of genetic divergence, haplotype network and Bayesian assignment analysis were consistent in rejecting the hypothesis of panmixia. *C*. *montagui* is genetically structured in three geographically discrete populations, which corresponded to north-eastern Atlantic Ocean, western-central Mediterranean Sea, and Aegean Sea-Black Sea. These populations are separated by two main effective barriers to gene flow located at the Almeria-Oran Front and in correspondence of the Cyclades Islands. According to the ‘isolation with migration’ model, adjacent population pairs diverged during the early to middle Pleistocene transition, a period in which geological events provoked significant changes in the structure and composition of palaeocommunities. Mismatch distributions, neutrality tests and Bayesian skyline plots showed past population expansions, which started approximately in the Mindel-Riss interglacial, in which ecological conditions were favourable for temperate species and calcium-uptaking marine organisms.

## Introduction

The understanding of current and historical factors that shape species’ genetic structure in broadcast spawning coastal marine invertebrates is an intriguing topic. Most marine invertebrate species spend a part of their life cycle in the pelagic realm as free-moving gametes, larvae, juveniles or adults, providing moderate to high potential for dispersal and favouring genetic homogenisation even over large distances. As a consequence, the identification of population borders in marine broadcast spawning species is not an easy task. However, the tendency to genetic homogenisation can be counteracted by the occurrence of geographical and oceanographical barriers to dispersal that determine restriction to gene flow, promoting the onset of genetically differentiated populations [1].

In recent decades, population genetic studies of marine species highlighted biogeographical breaks due to physical or hydrographical barriers that restrict the dispersal. In the Mediterranean basin the main biogeographical break is the Almeria-Oran oceanographical front, located at about 300 kms east of Gibraltar at the Atlantic-Mediterranean interface [2]. Other breaks have been observed in the Siculo-Tunisian Strait, separating Western and Eastern Mediterranean Sea; the Otranto Strait, separating the Adriatic Sea from the Ionian Sea; the isolation of the Aegean Sea due to the particular hydrological conditions of this area [3] and lastly, the system of the Turkish straits separating the Aegean Sea from the Black Sea. Since the late Miocene, these barriers underwent the effects of the geological history of the region, in which two main events played important roles: (*i*) the Messinian Salinity Crisis, that separated the Mediterranean Sea from the Atlantic Ocean [4] and (*ii*) the dramatic Pleistocenic events with the periodical succession of long glacial and short interglacial periods [5].

An effective contribution to the phylogeography and identification of population borders in broadcast spawning species is provided by mitochondrial DNA (mtDNA) markers that in the last decades were largely employed [1,2,6]. Despite the high potential for dispersal of these species, several instances of genetic structuring were identified at the Atlantic-Mediterranean border and within the Mediterranean Sea [2]. Pleistocenic events that caused temporary constraints or even isolation of water bodies and present-day oceanographical barriers are often invoked to explain the observed patterns of genetic divergence. In addition, molecular markers offer the possibility to infer on historical factors that affected population structure [1].

*Chthamalus montagui* Southward 1976 is a barnacle found on the intertidal rocky shores of the NE Atlantic, from NW Scotland to Senegal, of the northern coasts of the Mediterranean Sea and Black Sea [7–10]. It is a hermaphroditic species that may self-fertilise in isolated conditions [11]. In the Atlantic, the species is characterised by a planktotrophic larval life-span of 14–17 days, whose duration depends on temperature and nutrients availability [12], but no data are available for the Mediterranean Sea. Differently to adult individuals of other barnacles, which passively disperse through rafting and ship-mediated transport, adults of *C*. *montagui* cannot travel on floating objects, as they require alternate exposure to air and water, a condition available only in the “surf-and-swash” zone of rocky shores [13].

The genetic structure of *C*. *montagui* was investigated for the first time by Dando et al. [14] and subsequently by Dando & Southward [15], Pannacciulli et al. [8] and Shemesh et al. [10]. All these authors highlighted pronounced genetic structuring in *C*. *montagui* over a large geographical scale.

In the present study we intend to provide a deeper insight into the species’ genetic structure and demographic history by using a region of the mitochondrial *COI* gene, following the promising results obtained with this marker by Shemesh et al. [10] in *C*. *montagui* and by Cheang et al. [16], Wu et al. [17] and Govindarajan et al. [18] in other chthamalid species. It is worth noting that the work by Shemesh et al. [10] highlighted that *COI* was a marker much more informative than *ITS* and *EF-1* for *C*. *montagui*. In our study, particular attention is paid to (*i*) current issues related to the presence of geographical and oceanographical barriers to gene flow and (*ii*) historical factors that promoted divergence and changes in population size.

## Materials and methods

### Sample collection

**Fig 1:**
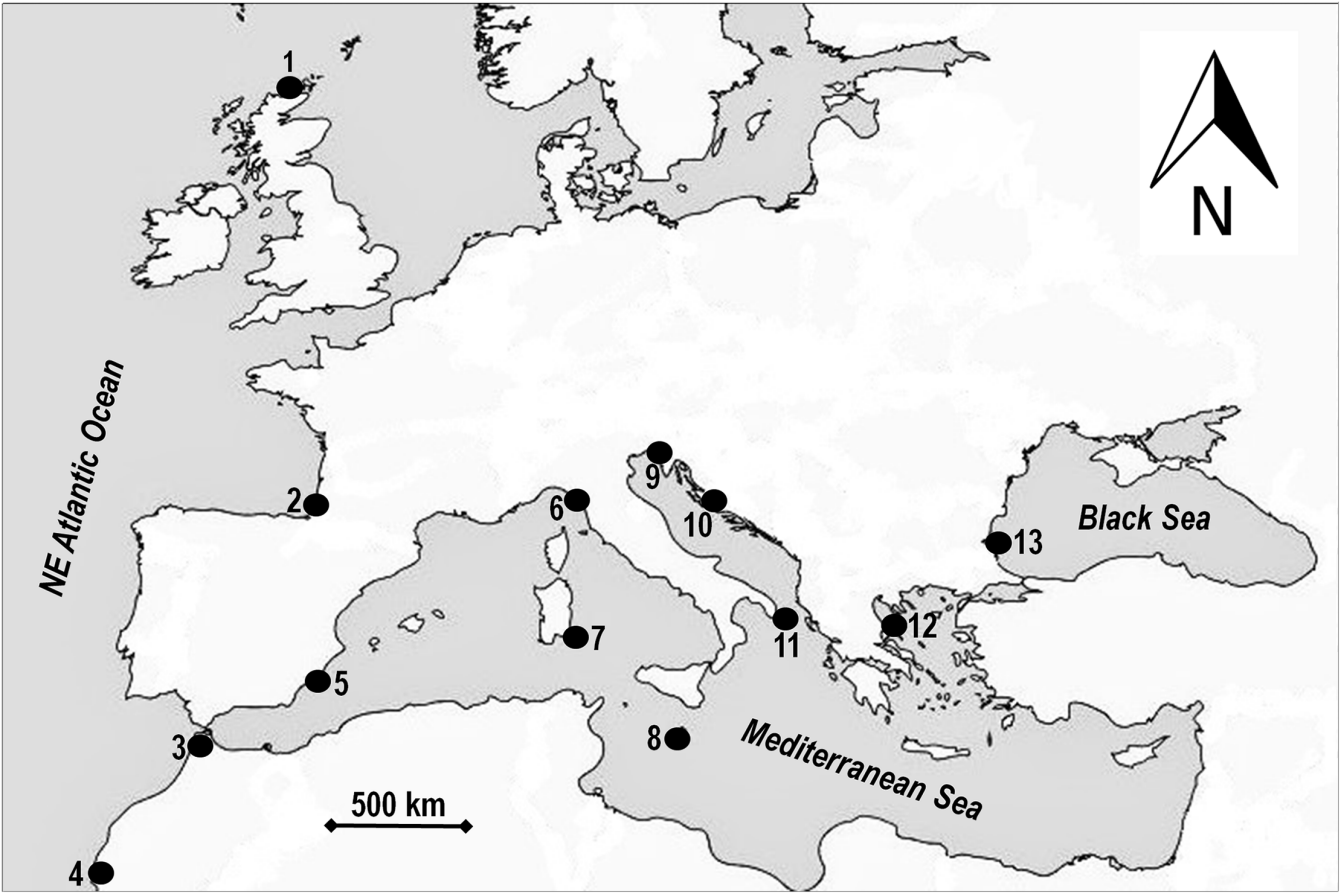
Location of *Chthamalus montagui* sampling sites. Location abbreviations are as reported in Table 1.

A total of 312 specimens of *Chthamalus montagui* were collected, between 2013 and 2014, from the intertidal zone at 13 localities across the known species’ geographical range (Fig 1). We sampled 24 individuals from each location, except for Agadir, Portman and Castro, where 25, 18, and 29 specimens were taken, respectively (Table 1). Specimens were removed from the substratum with a knife and fixed in 95% ethanol; after species’ identification through shell morphology, samples were stored at -20°C until DNA extraction. No specific permissions were required for sampling in these locations, the study did not involve endangered or protected species.

**Table 1:** *Chthamalus montagui*. **G**eographical coordinates of sampling locations and estimates of *COI* sample genetic diversity.

#	Location (Abbrev.)	Latitude	Longitude	*N*	*N*_h_	*N*_p_	*h ±*S.D.	π *±*S.D.
1	Skerray (SKE)	58°31'16"N	4°19'44"W	24	9	9	0.837 ±0.051	0.0029 ±0.0004
2	Biarritz (BIA)	43°28'48''N	1°34'12''W	24	11	11	0.851 ±0.054	0.0032 ±0.0005
3	Tangier (TAN)	35°47'38"N	5°49'28"W	24	14	17	0.913 ±0.016	0.0049 ±0.0006
4	Agadir (AGA)	30°21'05"N	9°35'29"W	25	19	17	0.967 ±0.024	0.0046 ±0.0006
	**North-Eastern Atlantic Ocean (NEA)**			**97**	**43**	**39**	**0.928** ±**0.016**	**0.0043** ±**0.0003**
5	Portman (PRT)	37°34'51"N	0°51'06"W	18	9	12	0.837 ±0.075	0.0075 ±0.0007
6	Baia Blu (BAI)	44°04'48"N	9°53'24"E	24	8	10	0.736 ±0.066	0.0064 ±0.0005
7	Cala Sinzias (CSZ)	39°11'24"N	9°34'12"E	24	14	18	0.931 ±0.033	0.0087 ±0.0009
8	Malta (MLT)	35°54'00"N	14°30'36E"	24	17	19	0.949 ±0.032	0.0143 ±0.0006
9	Grado (GRA)	45°40’29"N	13°23’08"E	24	14	20	0.888 ±0.057	0.0060 ±0.0011
10	Zaton (ZAT)	42°41'24"N	18°02'24"E	24	10	9	0.797 ±0.070	0.0034 ±0.0007
11	Castro (CAS)	40°01'47"N	18°27'03"E	29	10	16	0.756 ±0.079	0.0063 ±0.0012
	**Western-Central Mediterranean Sea (WCM)**			**167**	**66**	**50**	**0.877** ±**0.022**	**0.0088** ±**0.0006**
12	Volos (VOL)	39°20'24"N	22°56'24"E	24	11	18	0.750 ±0.092	0.0037 ±0.0011
13	Sozopol (SOZ)	42°25'12"N	27°42'00"E	24	11	10	0.819 ±0.073	0.0028 ±0.0005
	**Aegean Sea—Black Sea (ABS)**			**48**	**21**	**28**	**0.788** ±**0.062**	**0.0034** ±**0.0007**
	**TOTAL STUDY AREA**			**312**	**130**	**91**	**0.953 ±0.007**	**0.0188 ±0.0005**

*N*, sample size; *N*_h_, number of haplotypes; *N*_p_, number of polymorphic sites; *h*, haplotype diversity; π, nucleotide diversity.

### DNA extraction, amplification and sequencing

Individuals were removed from their shells and genomic DNA was extracted from the soft tissue using a proteinase K/salting out method [19]; DNA was stored in TE solution (1 M Trizma base, 0.5 M pH 8 EDTA) at -20°C until genetic analyses.

*COI* fragments were amplified by using the universal primers LCOI1490 and HCOI2198 [20]. Polymerase chain reaction (PCR) amplifications were carried out in 20 μl reactions using 1× PCR buffer, 1.25 mM of MgCl_2_, 0.2 mM of each dNTP, 1 μM of each primer and 0.01 U of *Taq* DNA polymerase. The PCR profile consisted in an initial 2-min denaturation at 95°C, 35 cycles of 90-sec denaturation, 30-sec annealing at 50°C, 45-sec at 72°C, and a final 10-min extension at 72°C. The PCR products were purified using ethanol precipitation [21] and shipped to Mc LAB (San Francisco, USA) for automatic sequencing by capillary electrophoresis.

### Sequence data analyses

Sequences were edited in Bioedit v. 7.2.5 [22] and aligned with ClustalX v. 2.1[23]. As reference, we used a *COI* sequence of *C*. *montagui* downloaded from GenBank (accession number FJ858066.1). Each haplotype found in the present work was deposited in GenBank (accession numbers KU682059 to KU682188). A hierarchical array of tests based on the Bayesian Information Criterion was carried out with jModelTest v. 2 [24], in order to identify the most appropriate nucleotide substitution model among 88 models. Haplotype diversity (*h*) and nucleotide diversity (π) were calculated with DnaSp v. 5.10.01 [25].

Hereafter we consider the following three biogeographical areas based on results of preliminary analyses of variance (AMOVA), haplotype network, and Bayesian assignment test (see below and Results section): North-eastern Atlantic Ocean (NEA), Mediterranean Sea with the exception of Aegean Sea (WCM), and Aegean Sea + Black Sea (ABS).Two AMOVAs [26] were carried out to examine the partition of genetic variance into (*i*) the “among-location” and “within-location” components and (*ii*) the “among-area”, “among-location within area”, and “within-location” components. The significance of Φ-statistics parameters was assessed by permutation tests with 10000 replicates as implemented in Arlequin v. 3.5.1.2 [27]. Estimates of genetic divergence (*F*_ST_) and gene flow (*N*m, number of effective migrants per generation [28]) between areas were obtained with DnaSp.

Mantel test (1967) with 10000 permutations was applied to the matrices of pairwise *F*_ST_-values and between-location minimal nautical distances to assess the presence of isolation by distance. The software Isolation by Distance Web Service [29] was employed for this purpose.

A median-joining network of haplotypes was obtained using Network v. 4.6.1.3 [30]. The software BAPS (Bayesian Analysis of Population Structure [31,32]) was employed giving a deeper insight into the genetic structure by grouping genetically similar individuals into panmictic genetic clusters. BAPS was set with six replicate runs for each value of *k* (the maximum number of genetic cluster) up to *k* = 20. In addition, we set a number of reference individuals *n* = 200 and repeated the admixture analysis 20 times for individual.

We used the Isolation with Migration analytic (IMa) program [33] to estimate the time of divergence between the NEA-WCM and WCM-ABS population pairs. IMa employs a Bayesian coalescent-based Markov Chain Monte Carlo (MCMC) approach to estimate the time of population divergence, which is expressed in number of generations and scaled by mutation rate. We specified a generation time of 1 year [34] and a substitution rate of 3.1% per million years for *COI*, according to Wares [35] and Tsang et al. [36]. After a number of initial trials, we set a geometric heating scheme with ten chains with the following upper bounds for each parameter: q1 = q2 = qa = 10, m1 = m2 = 10, t = 10) and performed three replicate runs. Each run consisted of a 5*10^5^ burn-in and 5*10^7^ MCMC iterations with genealogy sampling frequency of 10^4^.

For each area, the demographic history was inferred by mismatch distribution analysis [37] as implemented in Arlequin. The fit of our data with the expected model was evaluated with Schneider & Excoffier’s [38] bootstrap approach, using the sum of squared deviations (SSDs) between the observed and the expected mismatch distributions. In addition, Harpending’s [39] raggedness index (*r*) and Fu’s [40] *F*_S_ were computed to assess population expansion and tested with coalescent simulations with 10000 replicates, as implemented in DnaSp.

Furthermore, Beast v. 2.0 [41] was employed to infer historical demography through the Bayesian coalescent approach that improves recovery of the historical signal within DNA sequences. By using the Bayesian Skyline Plot (BSP) analysis [42], we estimated changes in effective population size over time for the three “biogeographical areas” detected through AMOVA and assignment tests (see Results section). BSPs were obtained assuming a piecewise constant model with ten coalescent intervals. MCMC simulations were run under the HKY+I model of nucleotide substitution using a relaxed molecular clock [42]. For each simulation, three independent replicate runs were carried out; MCMC chains were run for 5*10^7^ steps and sampled every 10^4^ steps. Ten percent of the sampled trees were discarded as burn-in. Tracer v. 1.6 [43] was used to assess convergence of runs through the effective sample size (ESS) of each parameter. In order to obtain an adequate ESS-value (≥200), the three independent runs performed for each simulation were combined using the LogCombiner v. 2.2.0 utility of the Beast package. The resulting file was used to estimate population size change through time, which was visualized by the Bayesian skyline plot obtained with the utility Tracer v. 1.6 (available at http://beast.bio.ed.ac.uk/Tracer).

## Results

### Within-sample genetic diversity

A total of 312 sequences, of a 537 bp portion of the *COI* gene of *Chthamalus montagui* were obtained across the 13 sampling localities. Overall, 130 haplotypes emerged from the sequence alignment, 98 of which were singletons (represented by only one individual), while 14 were shared. JModelTest [44] identified HKY+I [45] as the most probable evolutionary model with the following frequencies for each nucleotide base: f_A_ = 0.227; f_C_ = 0.166; f_G_ = 0.187; f_T_ = 0.420.

Sharing of haplotypes was not found among the above biogeographical areas (S1 Table). *COI* sequences were characterised by 91 (16.9%) polymorphic sites, a generally high haplotype diversity and low nucleotide diversity, with higher values only for Malta (Table 1).

Total haplotype diversity was *h* = 0.953 ±0.007, with the smallest value in the ABS area (*h* = 788 ±0.062) and the largest value in the Atlantic area (*h* = 0.928 ±0.016) (Table 1). The total nucleotide diversity was relatively high (π = 0.0188 ±0.0005), although at local scale π-values were an order of magnitude lower (π = 0.0029 ±0.0004 to 0.0087 ±0.0009), except for Malta which exhibited a value of π = 0.0143 ±0.0006 (Table 1).

### Among-sample genetic divergence

Considering the unstructured data set, AMOVA analysis showed significantly high molecular variance for the “among locations” component, accounting for 71.2% (Table 2). When we considered the hierarchical level “biogeographical area”, the AMOVA assigned a large portion of molecular variance to the “among-area” level (74.0%) that was similar to that given to the “among-location” component by the two-level analysis; the “among-location within area” and “within-location” levels accounted for 4.0% and 21.1%, respectively (Table 2). All Φ-statistics parameters associated to each level were highly significant (Table 2).

**Table 2:** *Chthamalus montagui*. Hierarchical AMOVA analyses for the *COI* fragment with no grouping and three biogeographical areas (1: SKE, BIA, AGA, TAN; 2: PRT, BAI, CSZ, MLT, CAS, ZAT, GRA; 3: VOL, SOZ). The significances of Φ-statistics values were tested by a permutation test with 10000 replicates.

Grouping criterion	Source of variation	df	% of variance	Φ-statistics
unstructured	Among locations	12	71.24	Φ_ST_ = 0.712*
	Within locations	299	28.76	
three areas	Among areas	2	74.90	Φ_CT_ = 0.749*
	Among locations within areas	10	4.03	Φ_SC_ = 0.161*
	Within locations	299	21.07	Φ_ST_ = 0.789*

* *P* < 0.001.

Pairwise *F*_ST_-values calculated between the three biogeographical areas indicated the occurrence of a high degree of genetic divergence between them, with the highest value detected, as expected, between the NEA and the ABS areas (Table 3). The *F*_ST_-based estimates of gene flow (*N*m) between the biogeographical areas were largely lower than 1, considered the threshold value under which two populations genetically diverge [46] (Table 3). Results of Mantel test, applied to the 13 geographical locations considered, were consistent with the isolation by distance (IBD) model in the study area, being the matrices of between-location *F*_ST_-values and geographical distances significantly correlated (*Z* = 25977.392, *P* = 0.006).

**Table 3:** *Chthamalus montagui*. Pairwise estimates of *COI* genetic divergence (*F*_ST_, below diagonal) and gene flow (*N*m, above diagonal) between the three biogeographical areas. The significances of *F*_ST_-values were tested by a permutation test with 10000 replicates.

	North-Eastern Atlantic Ocean	Western-Central Mediterranean Sea	Aegean Sea-Black Sea
North-Eastern Atlantic Ocean	0	0.161	0.064
Western-Central Mediterranean Sea	0.757*	0	0.339
Aegean Sea-Black Sea	0.886*	0.596*	0

* *P* < 0.05.

**Fig 2:**
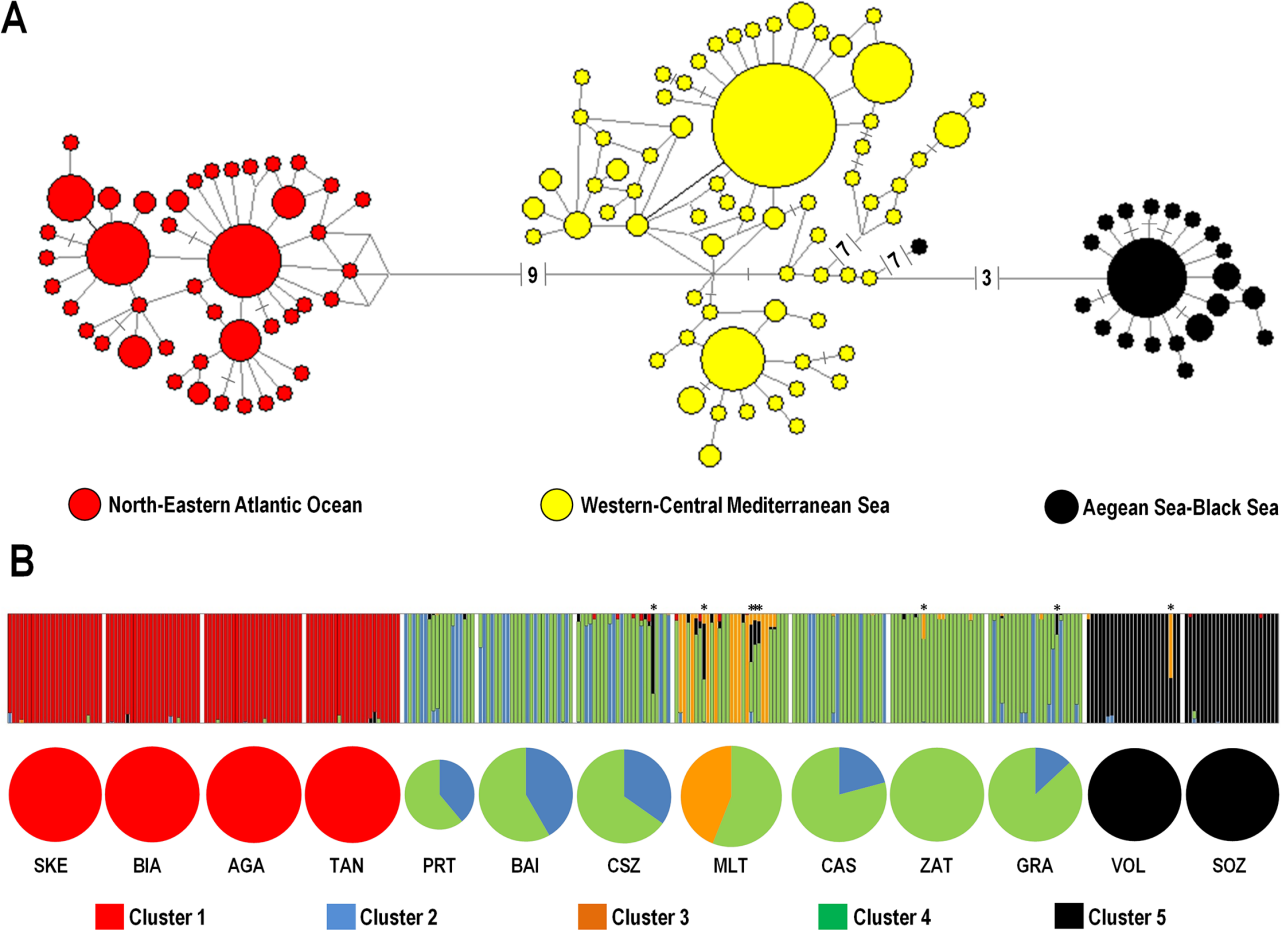
A. Median-joining network of *COI* haplotypes obtained in *Chthamalus montagui* from the 13 sampled locations. Each line in the network represents one mutational step; each small bar on the branches represents an additional mutational step. The area of each circle is proportional to the number of individuals showing that haplotype. B. Bayesian assignment analysis of *COI* sequences. Each vertical bar represents an individual and its associated probability of belonging to one of the five genetic clusters detected. Asterisks on the bar graph indicate individuals with uncertain assignment (*P* < 0.05). Pie charts indicate the percentage of genetic clusters contributing to each location. Pie charts were constructed with individual net assignments and after removing individuals with uncertain assignment.

The median-joining network of *COI* haplotypes revealed the presence of three consecutively connected sub-networks, each corresponding to a biogeographical area (Fig 2). The NEA sub-network was separated by nine mutational steps from the WCM one, which in turn was distant three mutational steps from the ABS one (Fig 2). Star phylogeny patterns were present within each area.

The Bayesian assignment test revealed the presence of five genetic clusters (GCs) with associated posterior probability, *PP* = 1 and eight instances of uncertain assignment (Fig 2). In each of the NEA and ABS areas only one genetic cluster was found (GC1 and GC5, respectively), whereas the remaining three genetic clusters (GC2, GC3, and GC4) were observed within the WCM, being the GC3 private to the Malta location; in addition, Malta and Zaton lacked of the GC2 (Fig 2).

The analyses to estimate the timing of population divergence carried out using IMa yielded consistent results across the three replicates and gave very similar dating values for both biogeographical area pairs. The NEA-WCM and WCM-ABS divergence times were 903 [95% highest posterior density interval (HPD): 475–1098] ky and 892 [95% HPD: 598–1121], respectively.

### Historical demography

**Fig 3:**
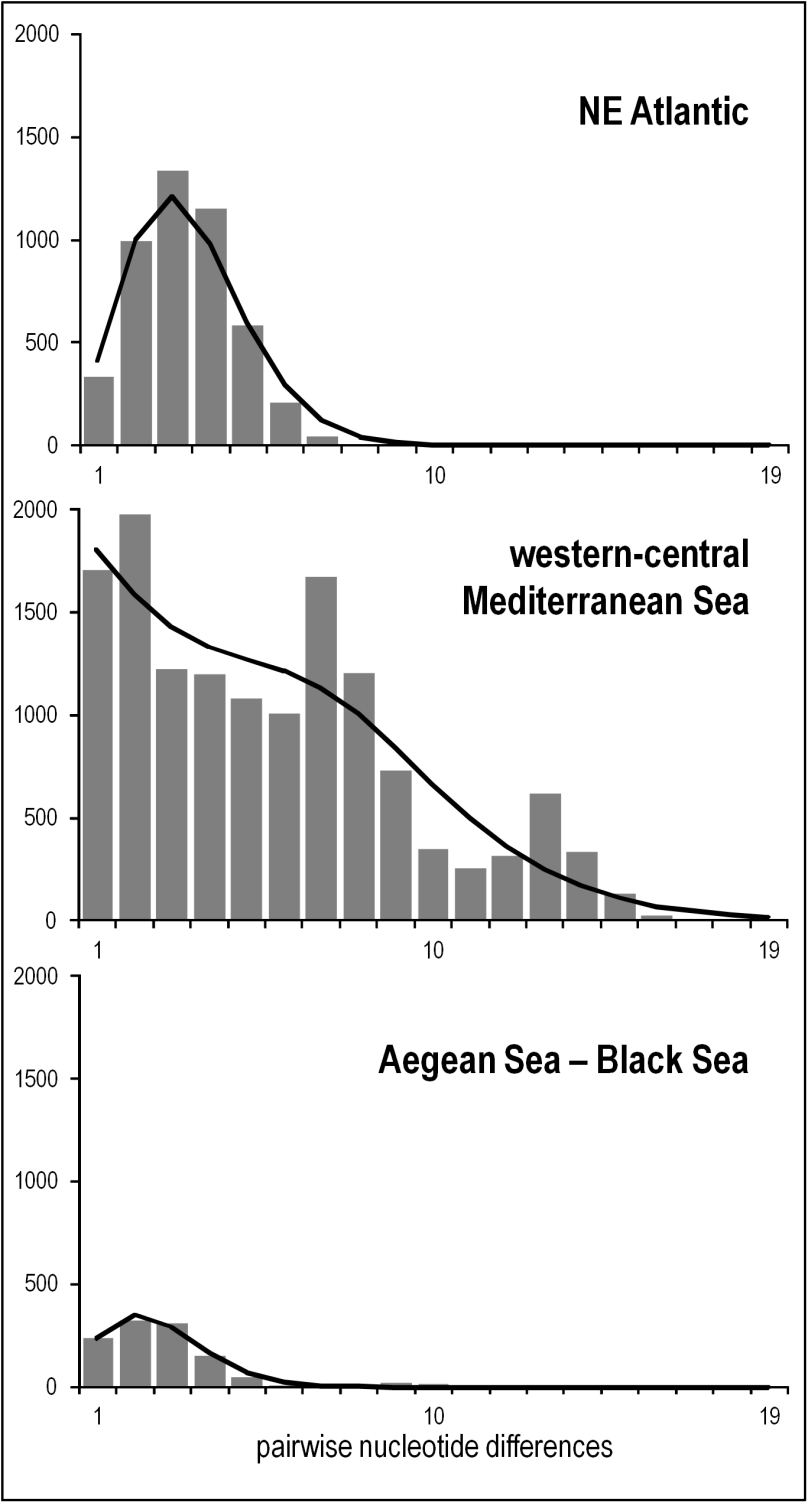
Frequency distribution of the number of pairwise nucleotide differences (mismatch) between *COI* haplotypes in the three populations of *Chthamalus montagui*. The solid line is the theoretical distribution under the model of demographic expansion.

The mismatch distributions showed a clear unimodal trend in the NEA and ABS areas, with curves that did not significantly deviate from Rogers & Harpending’s [37] model of recent demographic expansion (Fig 3, Table 4). This result was corroborated by significance of *r* and *F*_S_ indices (Table 4). Conversely, the mismatch distribution of the WCM was multimodal (Fig 3), even though the SSD from the expected curve was not significant (Table 4). The *F*_S_ index was consistent with this result while *r* was not (Table 4), indicating a controversial outcome for this area.

**Table 4:** *Chthamalus montagui*. Sum of squared deviations (SSD) between the expected and observed mismatch distributions of pairwise differences, Harpending’s [38] raggedness index (*r*) of the observed mismatch distribution and Fu’s [39] *F*_S_ neutrality test on *COI* sequences pooled according to the three biogeographical areas; *P* values were obtained by coalescent simulations with 10000 replicates.

	SSD	*r*	*F*_S_
north-eastern Atlantic Ocean	0.003^ns^	0.050^ns^	-52.942^**^
western-central Mediterranean Sea	0.005^ns^	0.010^*^	-62.957^**^
Aegean Sea-Black Sea	0.002^ns^	0.036^ns^	-18.596^**^

^*^ *P* < 0.05

^**^ *P* < 0.001

^ns^ not significant.

**Fig 4:**
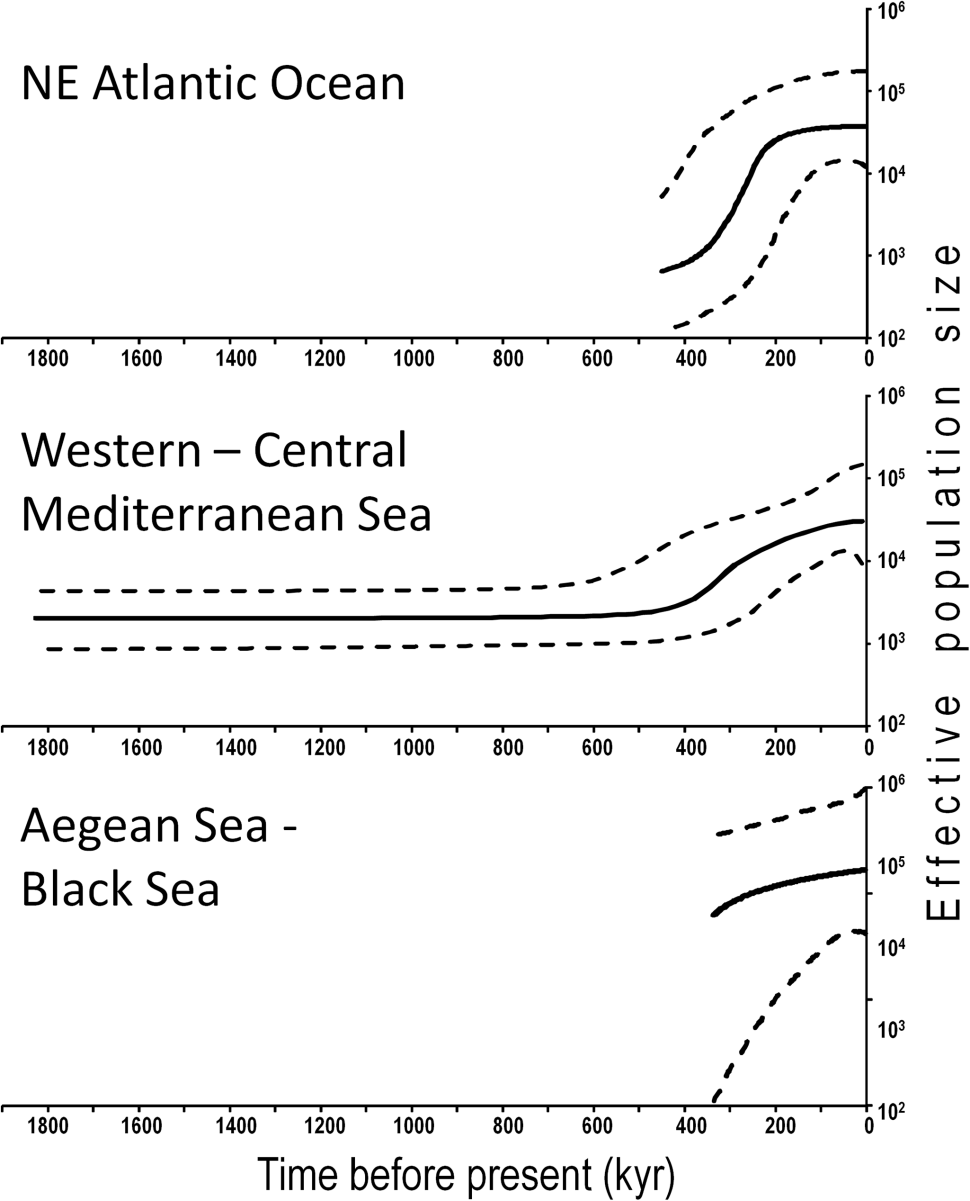
Bayesian skyline plots of effective population size through time in *Chthamalus montagui* from the three biogeographical areas, based on the 537bp sequences of *COI* and a nucleotide substitution rate of 3.1%/my. The bold black curve is the median of the parameter *N*_e_*T*, which is proportional to the effective population size; the dotted lines delimit the 95% highest posterior density. For comparison, all *x*-axes have the same scale. The plots are truncated to the median estimate of each area’s TMRCA.

The BSP analysis produced three curves consistent with a past event of demographic expansion (Fig 4). In the NEA and WCM areas, BSPs showed that demographic expansions started approximately 450 ka (Fig 4). The lower number of sequences available for the ABS, produced a plot that showed a tendency to demographic expansion, but was truncated at 430 ka; as a consequence, it was not possible to date precisely the beginning of population expansion for this area.

## Discussion

### Genetic structure

The analysis of the 312 *COI* sequences of *Chthamalus montagui* provided a useful contribution to the understanding of species’ genetic structure in its known geographical range. *COI* proved to be effective in providing a solid and informative approach for this phylogeographical survey. The high number of location-private haplotypes found in this study was high (98 of 130, corresponding to the 75%) and consistent with those observed in previous work on chthamalids with wide geographical distribution, such as *C*. *stellatus* (37 of 49, 76%, [10]) and *C*. *challengeri* (248 of 312, 79%,[16]).

The deep genetic structuring observed in *C*. *montagui* in the study area had a clear biogeographical meaning, with individuals grouped in three main genetic clusters that corresponded to north-eastern Atlantic (NEA), western-central Mediterranean Sea (WCM) and Aegean Sea-Black Sea (ABS). This result corroborated the three *COI* haplogroups found by Shemesh et al. [10]. As previously observed by Dando et al. [14], Dando & Southward [15], Pannacciulli et al. [8], and Shemesh et al. [10], the WCM area exhibited further genetic structuring, even though at a shallower degree. In the present study, the amount of genetic divergence between areas was surprisingly high. Accordingly, between-area estimates of gene flow were abundantly below *N* = 1, threshold value under which, as a rule of thumb, isolation between populations occurs [46]. An interesting finding of the present work relates to the presence of isolation by distance (IBD) among the three identified populations and not among samples within populations, despite the large spatial scale covered (e.g. the four Atlantic locations span over 3380 km). It should be noted, in fact, that for detecting IBD in broadcast spawning species, a spatial scale 2–5 times wider than the distance of the average larval dispersal is required [47]. Given that the maximum dispersal distance for *Chthamalus* spp. was estimated to be approximately 200 kilometres [48,49], the spatial scale adopted in this study is appropriate. However, when interpreting results on IBD in *C*. *montagui*, two additional factors should be taken into account. Firstly, we cannot exclude the occurrence of false positivity of this test due to the presence of significant genetic structuring [50]. Secondly, in marine broadcast spawning species, IBD can go undetected because the ‘realised geographical distance’, namely the distance adjusted by oceanographical and behavioural factors could largely differ from the actual nautical distance [51].

### The Atlantic/Mediterranean break

Dando and Southward [15] firstly argued that historical factors, combined with current hydrographical patterns, might have promoted and maintained differentiation between Atlantic Ocean and Mediterranean Sea populations of *C*. *montagui*. The Messinian closure of the Strait of Gibraltar (around 5.6 Ma), which determined extensive desiccation of the Mediterranean basin [4], is often taken into account to explain the differentiation between marine populations of the two basins. The modern Mediterranean marine fauna originated from organisms that entered the Mediterranean Sea after the re-opening of the Strait of Gibraltar (approximately 5.3 Ma), with the exception of a number of euryhaline relicts that survived in refugia. Our results suggest that more recent historical factors accounted for the Atlantic-Mediterranean divergence in *C*. *montagui* (see also the paragraph ‘Timing of divergence between populations’).

Nevertheless, we argue that two other factors may have produced and maintained the observed genetic divergence. Firstly, as observed in previous works [8,10,14,15], hydrographical factors, such as the Almeria-Oran front (AOF), play an important role in restricting gene flow in both directions, preventing genetic homogenisation between Atlantic and Mediterranean populations of *C*. *montagui*. Dando and Southward [15] argued that the changeover between the Atlantic and Mediterranean “forms” of *C*. *montagui* can be placed in proximity of the AOF. This is the reason why we chose to analyse a sample from Portman, located in the eastern proximity of the AOF. Portman sample exhibited typical Mediterranean *COI* haplotypes, whereas the individuals from Tangier, located in correspondence of the Strait of Gibraltar, were characterised by the Atlantic ones (Fig 2; S1 Table). The genetic break generated by the AOF is classically reported for many marine species with Atlantic-Mediterranean distribution (reviewed in [2]), even though exceptions exist as, for instance, the deep-water blue-red shrimp *Aristeus antennatus* [52], which presents a genetic break in correspondence of the Strait of Gibraltar.

Secondly, the different ecological conditions of the Atlantic Ocean and Mediterranean Sea coastal habitats could generate different adaptive requirements for *C*. *montagui*, promoting genetic divergence even in presence of gene flow, as observed in several marine invertebrates (i.a. [51] and references therein). For instance, differences in tidal range, which imply different time of exposure to air, may play a very important role in generating area-specific ecological requirements leading to genetic divergence.

### The western and central Mediterranean Sea

The three genetic clusters observed in samples of *C*. *montagui* within this area reflect the presence of shallow genetic structuring. The exact mechanisms that generated this pattern cannot be directly inferred from our *COI* results, although the observed structure has a high degree of correspondence with the classical biogeographical sectors proposed by Bianchi & Morri [53] and Bianchi [3]. Explanations could take into account an ancient allopatric origin of the genetic clusters, followed by secondary contact favoured by the efficient larval dispersal.

Our results showed that the Strait of Sicily, namely the geographical border between the western and eastern Mediterranean Sea, and the Strait of Otranto, separating the Adriatic from the Ionian Sea, do not represent effective barriers to gene flow in *C*. *montagui*. For instance, in the latter case individuals from Castro, located in the northern Ionian Sea, displayed a genetic pattern similar to that of the Adriatic samples.

Unique genetic characteristics had already been observed in individuals from Malta and were attributed to local environmental conditions [8,54]. The occurrence of two genetic clusters in the Maltese sample, one shared with the rest of the western-central Mediterranean Sea, the other one uniquely found in Malta, opens the ground to further interpretations. Barnacles assigned to the genetic cluster private to Malta could be viewed as remnants of a relict population. Currently, Malta could act as a “sink location” for *C*. *montagui*, by receiving larvae from external sources and generating larvae that remain within the Bay of La Valletta, whose topographical and hydrographical conditions favour larval retention. Notably, this Bay is the only place of Maltese archipelago where *C*. *montagui* was found after extensive search (F.G. Pannacciulli pers. obs.). The relatively short duration of the larval stage of *C*. *montagui*, coupled with the dislike for very hydrodynamic environments typical of islands [7], make larvae of this species unable to reach distant rocky shores or settle on highly hydrodynamic coasts; as a consequence *C*. *montagui* is not present on islands [7]. However, during the Pleistocenic glaciations, the emerged land masses connecting the Maltese archipelago with Sicily [55], may account for the current presence of *C*. *montagui* on Malta.

Finally, the option of species’ passive transport through rafting and shipping could be excluded, as *C*. *montagui* needs regular exposure to air to consolidate shell plates [13]. On the other hand, transport of larvae in bilge waters is theoretically possible but no data are available on the mechanism nor on the recruitment success.

### The break between western-central Mediterranean Sea and Aegean Sea

As we reported for the Atlantic-Mediterranean break, the finding of the ABS cluster of *C*. *montagui* can be interpreted as a result of the interplay of historical and current events. During the Pleistocene, the Black Sea was subject to periodical isolation and partial desiccation. In the glacial periods, it became an isolated freshwater basin, due to the separation from the Mediterranean Sea and the input of the great continental rivers; in contrast, during the interglacial transgressions it was repeatedly colonised by the Mediterranean marine fauna [56–58]. *C*. *montagui* could have not survived in glacial Black Sea environment, so undergoing recurrent extinction-recolonisation phenomena in this basin. The ABS lineage observed in this study could have originated from coastal refugia of the Aegean Sea where these barnacles survived during the Last Glacial Maximum. The current distribution of *C*. *montagui* in the Black Sea derives from the last refilling and recolonisation of the basin, which occurred approximately 7 ka [59].

The isolation of the ABS lineage of *C*. *montagui*, in agreement with what observed by Shemesh et al. [10], could be maintained by the hydrographical features of the Aegean Sea, such as the predominant unidirectional surface currents entering from the Black Sea [60]. These currents generate a main surface water-flow along the Cyclades Islands towards the Levantine basin [61] that acts as a barrier to the southward larval dispersal towards the Mediterranean Sea and *vice versa*. In addition, an anticyclonic front present in the south-western part of the Peloponnese peninsula [62] could further restrict larval exchange. From a biogeographical perspective, this front is responsible of a break separating the Aegean populations from the Mediterranean Sea ones [3]. Isolation of the Aegean population was found in a number of marine invertebrates, such as the cockle *Cerastoderma glaucum* [58] and the cuttlefish *Sepia officinalis* [63].

Despite the much lower salinity and the wider range of variation of the surface temperature of the Black Sea compared to the Aegean Sea, no genetic divergence was found between the samples of *C*. *montagui* from this area. This outcome suggests that differences in salinity and water temperature do not play an important role in shaping species genetic architecture.

### Timing of divergence between populations

The very close estimates of divergence time obtained for the two population pairs (approximately 900 ka) lays between the lower and middle Pleistocene, more precisely in correspondence of the Calabrian-Ionian transition, which was characterised by magnetic reversal and switch from the dominance change of the 40 ky glacial/interglacial cycles to the 100 ky ones [64]. From a palaeozoological perspective, this transition corresponds to the terrestrial faunal “revolution”, which gave rise to a progressive reconstruction of mammalian faunal complexes [5]. Moreover, the particular astronomical configuration of the last node of 1.2 Ma obliquity cycles, centred at around 900 ka, led Bertini et al. [5] to hypothesise a significant influence of these events on the structure and composition of palaeocommunities. Our results suggest that these events may have played a role in shaping the genetic architecture of *C*. *montagui*, even though it is difficult to suggest mechanisms that underlie divergence between the two population pairs.

### Historical demography

Overall, starbust phylogenies in the haplotype network, mismatch distributions and neutrality tests showed that populations of *C*. *montagui* underwent past demographic expansion [1,37] (Figs 2A and 3; Table 4). The observed discrepancy between the three-peak mismatch distribution recorded in WCM and the unimodal curve in the other two biogeographical areas (Fig 3), is only apparent. In fact, although for WCM we observed a significant value of the raggedness index, the mismatch distribution did not significantly deviate from the expected curve. Multimodal distributions are generally interpreted as a signature of long term population stability [37]; however, in our case, the three peaks emerging from the mismatch distribution analysis were produced by population substructuring (the three genetic clusters observed within WCM) rather than demographic stability (Fig 3), as suggested by Aris-Brosou & Excoffier [65]. This outcome also accounted for the discrepancy observed between test of mismatch distribution (SSD) and Fu’s [39] *F*_S_ test. The latter implying population expansion and the *r* index suggesting, contrarily, demographic stability (Table 4).

The Bayesian Skyline Plot analysis (BSP) displayed the entire expansion curve for the NEA and WCM areas and a truncated curve for the ABS (Fig 4). Despite this truncation, due to the smaller sample size for the ABS, the curve shape was consistent with those of the other two areas. Results showed that the demographic expansion of *C*. *montagui* in the NEA and WCM areas started approximately 450 ka and continued for about 150–200 kyr (Fig 4). This dating corresponds to the “Marine Isotope Stage 11” (MIS11: 362–423 ka) that approximately matches the Mindel-Riss interglacial period [66]. MIS11 was a particular long-lasting interglacial in which orbital geometry of the Earth was very similar to that of the Holocene and a peculiar interplay between sea temperature, thermohaline circulation, plankton ecology, sea level, and reef growth was present. It is noteworthy that population expansion in the MIS11 was registered by BSP analysis for another barnacle, *Balanus glandula*, in the north-eastern Pacific [67].

During the MIS11, the increase of sea-water temperature could have triggered population expansion of *C*. *montagui*. This statement is also supported by results from several investigations carried out on modern populations, in which an increment in abundance was observed following the warming from the late 1980s [34,68,69]. Moreover, Burrows et al. [70] and O'Riordan et al. [71] found that high temperatures promote the release of a higher number of broods and increase recruitment rate, favouring an increment in population size.

Howard [66] also argued that in MIS11 palaeoecological conditions, such as the increase in sea-level and water temperature, triggered the biological production of calcium carbonate. These conditions could be an additional factor favouring the observed demographic expansion of *C*. *montagui* that requires large amounts of calcium carbonate for the construction of its robust shell.

## Supporting information

S1 Table *COI* haplotypes found in *Chthamalus montagui*. Distribution of the 130 haplotypes across locations and biogeographical areas.
(PDF)
