# Supplementary material for: Phylogeography on the rocks: The contribution of current and historical factors in shaping the genetic structure of Chthamalus montagui (Crustacea, Cirripedia)
Source: PLoS One. 2017 Jun 8;12(6):e0178287. doi: 10.1371/journal.pone.0178287 (PMC5464549; doi:10.1371/journal.pone.0178287)
Supplement: S1 Table — Distribution of the 130 haplotypes across locations and biogeographical areas. (PDF) [file pone.0178287.s001.pdf]

Haplotype number	GenBank accession number	Skerray	Biarritz	Agadir	Tangier	Portman	Baia Blu	Cala Sinzias	Malta	Grado	Zaton	Castro	Volos	Sozopol	NE Atlantic	WC Mediterranean	Aegean S. - Black S.
1	KU682059	1													1		
2	KU682060	5	8	2											15		
3	KU682061	2													2		
4	KU682062	1													1		
5	KU682063	8													8		
6	KU682064	1	1												2		
7	KU682065	4	5	4	6										19		
8	KU682066	1													1		
9	KU682067	1													1		
10	KU682068		3		1										4		
11	KU682069		1												1		
12	KU682070		1												1		
13	KU682071		1												1		
14	KU682072		1												1		
15	KU682073		1												1		
16	KU682074		1												1		
17	KU682075		1												1		
18	KU682076			1	1										2		
19	KU682077			1											1		
20	KU682078			1											1		
21	KU682079			1											1		
22	KU682080			1											1		
23	KU682081			1											1		
24	KU682082			1											1		
25	KU682083			1											1		
26	KU682084			1											1		
27	KU682085			1											1		
28	KU682086			1											1		
29	KU682087			1	1										2		
30	KU682088			1											1		
31	KU682089			3	3										6		
32	KU682090			1											1		
33	KU682091			1											1		
34	KU682092			1											1		
35	KU682093				4										4		
36	KU682094				1										1		
37	KU682095				1										1		
38	KU682096				1										1		
39	KU682097				1										1		
40	KU682098				1										1		
41	KU682099				11										11		
42	KU682100				1										1		
43	KU682101				1										1		
44	KU682102					3	8	1		1		2				15	

Haplotype number	GenBank accession number	Skerray	Biarritz	Agadir	Tangier	Portman	Baia Blu	Cala Sinzias	Malta	Grado	Zaton	Castro	Volos	Sozopol	NE Atlantic	WC Mediterranean	Aegean S. - Black S.
45	KU682103					7	10	5	1	8	10	14				55	
46	KU682104					1										1	
47	KU682105					1										1	
48	KU682106					2										2	
49	KU682107					1										1	
50	KU682108					1										1	
51	KU682109					1										1	
52	KU682110					1										1	
53	KU682111						1									1	
54	KU682112						1									1	
55	KU682113						1									1	
56	KU682114						1					1				2	
57	KU682115						1									1	
58	KU682116						1									1	
59	KU682117							1								1	
60	KU682118							1								1	
61	KU682119							2								2	
62	KU682120							2								2	
63	KU682121							4		2	5	3				14	
64	KU682122							2								2	
65	KU682123							1								1	
66	KU682124							1								1	
67	KU682125							1								1	
68	KU682126							1								1	
69	KU682127							1								1	
70	KU682128							1								1	
71	KU682129								1							1	
72	KU682130								1							1	
73	KU682131								5							5	
74	KU682132								3							3	
75	KU682133								1							1	
76	KU682134								1							1	
77	KU682135								2							2	
78	KU682136								1							1	
79	KU682137								1							1	
80	KU682138								1							1	
81	KU682139								1							1	
82	KU682140								1							1	
83	KU682141								1							1	
84	KU682142								1							1	
85	KU682143								1							1	
86	KU682144								1							1	
87	KU682145											2				2	
88	KU682146											1				1	

Haplotype number	GenBank accession number	Skerray	Biarritz	Agadir	Tangier	Portman	Baia Blu	Cala Sinzias	Malta	Grado	Zaton	Castro	Volos	Sozopol	NE Atlantic	WC Mediterranean	Aegean S. - Black S.
89	KU682147											3				3	
90	KU682148											1				1	
91	KU682149											1				1	
92	KU682150											1				1	
93	KU682151										1					1	
94	KU682152										1					1	
95	KU682153										1					1	
96	KU682154										1					1	
97	KU682155									1	2					3	
98	KU682156										1					1	
99	KU682157									1	1					2	
100	KU682158										1					1	
101	KU682159									1						1	
102	KU682160									1						1	
103	KU682161									1						1	
104	KU682162									2						2	
105	KU682163									2						2	
106	KU682164									1						1	
107	KU682165									1						1	
108	KU682166									1						1	
109	KU682167									1						1	
110	KU682168												1				1
111	KU682169												12	10			22
112	KU682170												1				1
113	KU682171												1				1
114	KU682172												1				1
115	KU682173												3				3
116	KU682174												1				1
117	KU682175												1				1
118	KU682176												1				1
119	KU682177												1				1
120	KU682178												1				1
121	KU682179													1			1
122	KU682180													1			1
123	KU682181													1			1
124	KU682182													2			2
125	KU682183													2			2
126	KU682184													1			1
127	KU682185													3			3
128	KU682186													1			1
129	KU682187													1			1
130	KU682188													1			1
